# Highly Toughened and Transparent Biobased Epoxy Composites Reinforced with Cellulose Nanofibrils

**DOI:** 10.3390/polym11040612

**Published:** 2019-04-03

**Authors:** Sandeep S. Nair, Christopher Dartiailh, David B. Levin, Ning Yan

**Affiliations:** 1 Faculty of Forestry, University of Toronto, 33 Willcocks Street, Toronto, ON M5S 3B3, Canada; sandeep.sudhakarannair@utoronto.ca; 2 Department of Biosystems Engineering, University of Manitoba, E2-376 Engineering and Information Technology Complex (EITC), 75 Chancellors Circle, Winnipeg, MB R3T 5V6, Canada; umdartia@myumanitoba.ca (C.D.); david.levin@umanitoba.ca (D.B.L.); 3 Department of Chemical Engineering and Applied Chemistry, University of Toronto, 200 College Street, Toronto, ON M5S 3E5, Canada

**Keywords:** biobased epoxy, cellulose nanofibrils, composite toughness

## Abstract

Biobased nanofillers, such as cellulose nanofibrils (CNFs), have been widely used as reinforcing fillers for various polymers due to their high mechanical properties and potential for sustainable production. In this study, CNF-based composites with a commercial biobased epoxy resin were prepared and characterized to determine the morphology, mechanical, thermal, and barrier properties. The addition of 18–23 wt % of CNFs to epoxy significantly increased the modulus, strength and strain of the resulting composites. The addition of fibrils led to an overall increase in strain energy density or modulus of toughness by almost 184 times for the composites compared to the neat epoxy. The addition of CNFs did not affect the high thermal stability of epoxy. The presence of nanofibrils had a strong reinforcing effect in both glassy and glass transition region of the composites. A significant decrease in intensity in tan δ peak for the epoxy matrix occurred with the addition of CNFs, indicating a high interaction between fibrils and epoxy during the phase transition. The presence of highly crystalline and high aspect ratio CNFs (23 wt %) decreased the water vapour permeability of the neat epoxy resin by more than 50%.

## 1. Introduction

Epoxy resins are a class of polymers that exhibit high strength, stiffness, and creep resistance, as well as dimensional and thermal stability. They have been widely used in coatings, as well as in composites, construction, adhesives, electronics, and electrical applications [1,2]. Most epoxy resins are derived from petroleum-based resources. Almost 75% of global production of epoxy resins are derived from the condensation reaction of bisphenol A (BPA) and epichlorohydrin, forming diglycidyl ether of bisphenol A (DGEBA). More than 60% of the molecular weight of DGEBA is petroleum-derived [3,4]. Growing environmental concerns, increased legislated obligations to move towards a sustainable economy, and volatile oil prices have all compelled both industry and academia to develop biobased epoxy resins. Therefore, a significant measure of research is committed to developing biobased epoxy resins or partially substituting the use of petroleum-based materials in synthesizing epoxy resin monomers. Several studies have focused on the preparation of epoxy resin monomers from various renewable materials such as tannins, cardanols, lignin, carbohydrates, vegetable oils, terpenes, and resin acids [1,2].

Despite all the good properties of biobased epoxy systems, they are inherently brittle. Epoxy resins fail under less vigorous conditions when compared to other engineering polymers due to its high crosslink density [2,5]. As a result, considerable research has been focused on improving the toughness of epoxy systems. Different ways have been used to improve the epoxy resin toughness. The major ones include (a) elastomer or rubber modification [6], (b) thermoplastic modification [7], (c) using inorganic rigid particles [8], and (d) miscellaneous methods, such as using dendritic molecules [9] or block copolymers [10]. However, most of these materials have their own limitations in toughening epoxy resins. Although, the addition of elastomers significantly increases toughness, it decreases the strength and stiffness [11]. Additionally, it is difficult to control the particle size of elastomers in the epoxy matrix [6]. The addition of thermoplastics increases ductility, including stiffness and strength. However, thermoplastics with high compatibility can negatively impact toughness due to non-phase separation [2,12]. Dendritic molecules or block copolymers are highly expensive [13]. In addition, most of these toughening agents are petroleum based, and/or non-renewable [2]. Over the years, scientists have developed different green approaches to overcome these difficulties. The major ones include developing epoxidized vegetable oils [14] and use of bio-based nanofillers such as CNFs [15] and cellulose nanocrystals (CNCs) [16] to toughen the epoxy.

Biobased nanofillers, such as cellulose nanofibrils (CNFs) have widths in nano-dimensions and high aspect ratio (length-to-width ratio). In plant fibers, several cellulose molecules are bundled together by strong hydrogen bonding to form elementary fibrils or microfibrils. These microfibrils or aggregates of microfibrils with diameters less than 100 nm are called as CNFs [17,18]. Intensive mechanical treatment is used to disintegrate or defibrillate plant fibers to nano-sized fibrils [19,20]. CNFs are abundant, biodegradable, renewable, and have unique physical properties. Modulus and strength as high as 3 GPa and 135 MPa, respectively, have been reported for some CNFs obtained from plant fibers [21,22]. The highly fibrillated CNFs have lesser defects than micro-sized cellulose fibers, and together with high aspect ratio can effectively increase the strength and strain of the reinforcing polymers, and thereby toughen the whole composite [18]. CNFs have been widely used as a reinforcing agent for a variety of thermoset resins [23,24] and thermoplastic polymers [25,26]. CNFs have also been studied as filler for various polymers for enhancing the water and oxygen barrier properties. High crystallinity, smaller dimensions, and the ability to form dense networks within the polymer are some of the significant barrier-enhancing properties of CNFs [17,27].

Several studies have reported the use of CNFs to enhance the mechanical properties of synthetic or petroleum based thermoset resin [23,24,28,29]. However, to the best of our knowledge, the addition of CNFs to enhance the mechanical properties of biobased epoxy resins has been very limited. In this study, our aim is to incorporate CNFs in a commercial biobased epoxy resin, to investigate the morphology and physical properties of the final composite material in terms of mechanical, thermal, and barrier properties.

## 2. Materials and Methods

### 2.1. Materials

The epoxy investigated is a commercial biobased epoxy resin, BioPoxy 36, supplied by Ecopoxy Inc. (Morris, MB, Canada). A medium hardener was the curing agent obtained from the same manufacturer. Cellulose nanofibril suspensions were obtained from the Center for Biocomposites and Biomaterials Processing, University of Toronto, Toronto, Canada. The cellulose content for the CNFs were 83% (based on dry weight), and the average fibril diameter was approximately 10–25 nm. The degree of polymerization and crystallinity for the CNFs were 1200 and 71%, respectively. All other chemicals were obtained from Caledon labs (Georgetown, ON, Canada).

### 2.2. Cellulose Nanofibril Sheet Preparation

CNF suspension at 0.1 wt % solid consistency was vacuum filtered through a membrane (0.22 µm PVDF, Millipore GVWP14250, MilliporeSigma, Bedford, MA, USA) in a Buchner funnel. The wet films obtained after dewatering the suspensions along with the membranes were placed between blotting papers and pressed at 345 kPa for 6 min each. Some of the films were removed and dried at 50 °C under a pressure of approximately 23 kg for two days. A separate batch of wet films was then solvent exchanged to acetone by soaking in acetone for 48 h. The dried as well as solvent exchanged films were cut into dog-bone shape tensile specimens according to ASTMD 638-Type V specifications.

### 2.3. Production of CNF/Epoxy Composites

The cellulose nanofibril network obtained by vacuum filtration was solvent exchanged with acetone before impregnating with epoxy. The solvent exchange is done to prevent aggregation of fibrils. The epoxy resin consisted of epoxy/curing agent mixed in a ratio of 4.29:1 by weight. To reduce the viscosity of resin, the epoxy resin was diluted with acetone in three different ratios: higher viscosity (epoxy and acetone at a weight ratio of 3:1); medium viscosity (1:1 ratio); and lower viscosity (1:3 ratio). Solvent exchanged CNF sheet specimens were immersed in each of these epoxy resin solutions. The impregnated specimens were removed from the solutions and suspended using fine metal wires in an oven for two hours prior to the curing. In addition to impregnated CNF specimens, neat epoxy films were solvent casted in aluminum pans. The specimens were then cured in the oven for 2 h at 120 °C. The cured samples were analyzed for various physical properties.

### 2.4. Mechanical Properties

The dried CNF films, neat epoxy films and composites were tested according to ASTM D 638-Type V specifications. Tensile tests were performed using an Instron machine (Model 3367, Instron, Norwood, MA, USA) equipped with a 2 kN load cell, in which a gauge length of 2.5 cm and a cross head speed of 10 mm min^−1^ were used. Dynamic mechanical analysis (DMA) were carried out using a TA Instruments Q800 analyzer (TA Instruments, New Castle, DE, USA) in tension mode. Rectangular test specimens were 6–7 mm wide and the span was 15 mm. The frequency and amplitude of oscillation were set at 1 Hz and 15 μm, respectively. The temperature varied from room temperature to 180 °C at a heating rate of 3 °C/min.

### 2.5. Thermogravimetric Analysis (TGA)

The thermal stabilities of the CNF films, neat epoxy films, and composites were obtained using a TGA Q500 (TA Instruments, New Castle, DE, USA) equipped with platinum crucibles. Samples (8−10 mg) were heated at a constant rate of 10 °C/min from room temperature to 800 °C in nitrogen atmosphere. The T_onset_ for the sample was defined as the temperature at which significant weight loss begins and determined using the tangent method. The T_max_, defined as the temperature at which the maximum weight loss rate occurred, corresponds to the maximum value of the derivative weight curve (dm/dT_max_).

### 2.6. Water Vapour Transmission (WVT)

WVT was determined according to ASTM E-96-95 water method. The test conditions were set to 23 °C and 50 % relative humidity (RH). The CNF films, neat epoxy and composite films were cut into 6.4 cm diameter specimens and conditioned to test conditions. The specimens were used to seal a test dish containing 50 mL of distilled water. The weight change of the test dish was recorded periodically and WVT was determined from the slope of weight change versus time, using the following equation:

WVT = ΔG/ΔtA(1)

Here, ΔG is the weight change of dish, Δt is the time during which the change occurred, and A is the test area (dish mouth area).

### 2.7. Other Characterizations

Scanning electron microscopy (SEM) images of the cross-sectional fracture surfaces of the CNF, epoxy and composite films were obtained using a JEOL 6610LV (Seal Laboratories, El Segundo, CA, USA) operated at 15 kV. Samples were mounted on an aluminum stub using carbon tapes and sputtered with gold to provide adequate conductivity. Attenuated total reflectance−Fourier transform infrared spectroscopy (ATR-FTIR) was used to obtain spectra of CNF film, neat epoxy, and composites ((ATR-FTIR, Nicolet iS50 FT-IR, ThermoFisher Scientific, Waltham, MA, USA). The sample spectra were collected from 600 to 4000 cm^−1^ at a resolution of 4 cm^−1^ after 64 scans. Transmittance spectra of the CNF, epoxy and composite films were obtained in the visible region (300–800 nm) using UV–VIS spectrophotometer (Lambda 2, PerkinElmer, Waltham, MA, USA).

### 2.8. Statistical Analysis

Twelve to fifteen specimens were tensile tested to obtain the average mechanical properties of pure CNF, neat epoxy and composite films. A graphical representation of the change in mechanical properties of composites with varying CNF weight percent will be obtained. Three specimens from each group (CNF, neat epoxy and composites films) will be analyzed for TGA, DMA, WVT, FTIR, and UV–VIS transmission spectra.

## 3. Results

**Figure 1:**
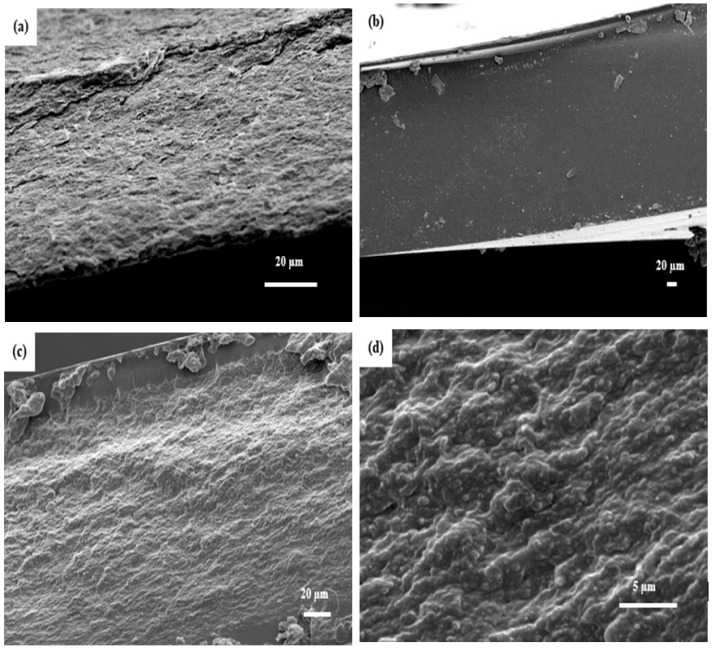
SEM images of fracture surfaces of (**a**) Pure CNF film; (**b**) neat epoxy film; (**c**) 23 wt % CNF/epoxy composite (low resolution); and (**d**) 23 wt % CNF/epoxy composite (high resolution).

**Figure 2:**
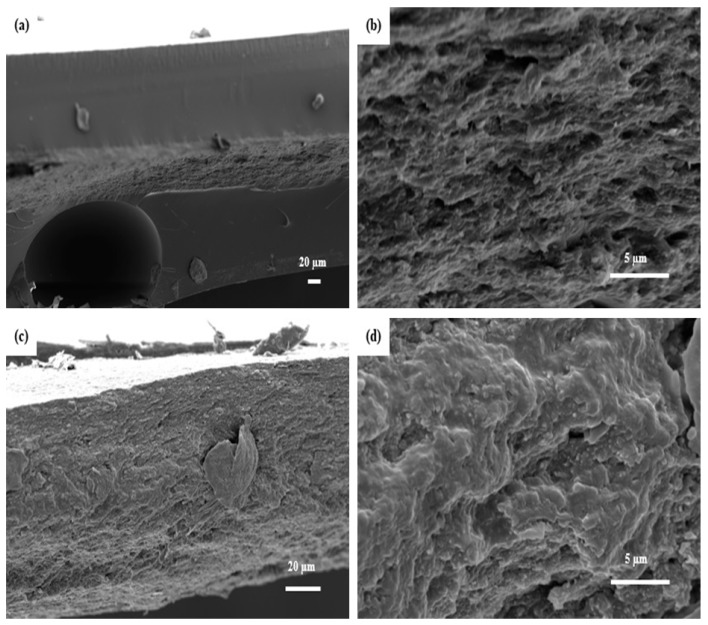
SEM images of fracture surfaces of cured composite made from impregnating CNFs in (**a**) epoxy/acetone solution at a ratio of 3:1 (low resolution); (**b**) epoxy/acetone solution at a ratio of 3:1 (high resolution); (**c**) epoxy/acetone solution at a ratio of 1:3 (low resolution); and (**d**) epoxy/acetone solution at a ratio of 1:3 (high resolution).

A series of composites were prepared by impregnating the pure CNF films in different epoxy/acetone solution ratios. The average thickness of CNF film ranged from 55–60 µm (Figure 1a). The impregnation of CNF films in medium viscosity (epoxy and acetone at a ratio of 1:1) yielded the best results. The composite cross-section showed well-dispersed fibrils or fibril aggregates within the epoxy matrix (Figure 1c,d). The white spots on the matrix are very short pull out lengths of fibrils or fibril aggregates indicating strong interfacial adhesion. The average thickness of the composites impregnated in medium viscosity ranged from 180–200 µm. However, the impregnation of CNF films in higher viscosity (epoxy and acetone at a ratio of 3:1) and lower viscosity (epoxy and acetone at a ratio of 1:3) did not yield good results due to the poor distribution of epoxy in and around the CNF fibrils. Figure 2a,b shows the cross section of composites obtained from impregnation in high viscosity epoxy solution. There was clear phase separation between the epoxy matrix and CNF film. Huge amount of epoxy formed the face layers, while the CNF film formed the core with dimensions similar to pure CNF film (50–70 µm). The total average thickness of the composite ranged from 290–320 µm. This implies that there was only limited penetration of epoxy into the CNF film. In addition, there were voids within the sample due to high viscosity of epoxy. For films impregnated in low viscosity solution (Figure 2c,d), the cross-sections showed non-uniform distribution of fibril within the matrix. There were areas totally devoid of any fibrils, sparse distribution of fibrils, and areas without any resin. The average thickness of composite ranged from 88–96 µm.

**Figure 3:**
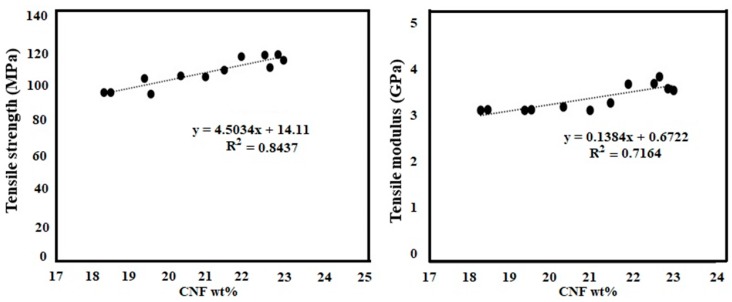
Mechanical properties of the CNF/epoxy composites.

**Figure 4:**
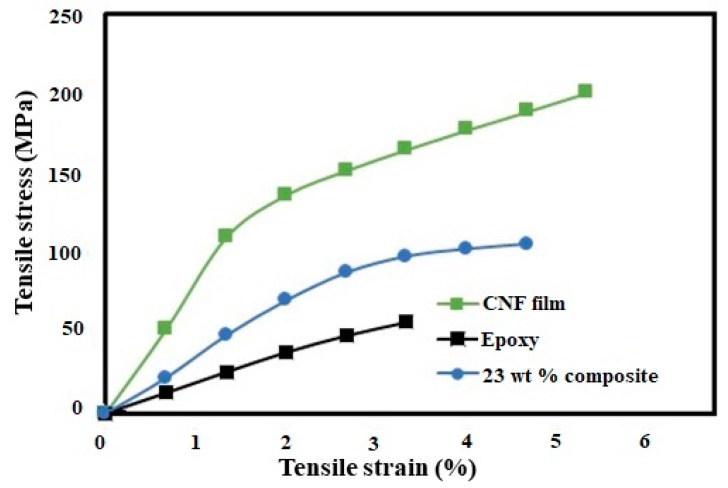
Representative stress-strain curves of the CNF film cured neat epoxy resin, and composite.

The medium viscosity led to good impregnation and resulted in composites with different CNF contents ranging from 18 to 23 wt %. Figure 3 shows the change in mechanical properties with varying CNF wt %. Table 1 shows the average mechanical properties of pure CNF film, neat cured epoxy resin, and the composite. The representative stress-strain curves for the tensile tests are shown in Figure 4. The strong reinforcement of CNF on the epoxy polymer matrix is quite evident from different loading of fibrils (18–23 wt %). The strain energy density or modulus of toughness was calculated as the total area under the stress strain curves. The modulus of the epoxy increased from an average value of 2.28 to 3.56 MPa, the tensile strength increased from 62.5 to 108.25 MPa, and the strain increased from 2.92% to 4.81%, which led to an overall increase in modulus of toughness by almost 184 times (0.98 to 2.79 MPa) for the composites compared to the neat epoxy.

Furthermore, the experimental data were compared with the predicted values using the rules of mixture (ROM). The predicted values were obtained using the following equations:

E_composite_ = V_f_E_f_ + (1 − V_f_) E_m_ (2)

σ_composite_ = V_f_σ_f_ + (1 − V_f_) σ_m_ (3)

Here, E_composite_ and σ_composite_ are the predicted modulus and tensile strength for the composites, respectively. E_f,_ E_m,_ σ_f,_ σ_m is_ the tensile modulus of CNF, tensile modulus of matrix, tensile strength of CNF, and tensile strength of matrix, respectively. V_f_ denotes the volume fraction of CNF. The tensile property of pure CNF film (E_f,_ σ_f_), neat epoxy (E_m,_ σ_m_) obtained from Table 1 were used to fit in the predicted model. The predicted strength and modulus values for 18 wt % loading (V_f_ = 13.7%) were 84.08 MPa and 3.02 GPa, respectively. The predicted strength and modulus values for 23 wt % loading (V_f_ = 17.77%) were 90.49 MPa and 3.24 GPa, respectively. The experimental values (Figure 3) for 18 wt % (E_composite_ = 3.31 GPa and σ_composite_ = 97.11) and 23 wt % (E_composite_ = 3.79 GPa and σ_composite =_ 118.7 MPa) were higher than the predicted values. 

Cellulose molecules show high polarity and, therefore, need chemical functionalization of the CNF to have a significant reinforcement effect on the epoxy matrix [30]. A good interaction between the CNF and epoxy can create an effective interphase region around fibrils. The stress in the interphase region will be higher than the neat epoxy, and could contribute to higher volume fraction of fibrils, thereby contributing to the high mechanical properties [31]. The interphase or the increase in volume fraction due to interphase is not taken into consideration for the ROM prediction model, which could result in lower predicted values. The results imply that the CNF used in this study showed good compatibility with the epoxy matrix, resulting in high mechanical properties. The composite tensile properties obtained in this study were comparable to, or even better than, some of the petroleum-based epoxy/CNF composites in the literature [28,29,32]. The reinforcement obtained by the addition CNF were even better than some of the glass fiber reinforced epoxy composites [33,34].

**Figure 5:**
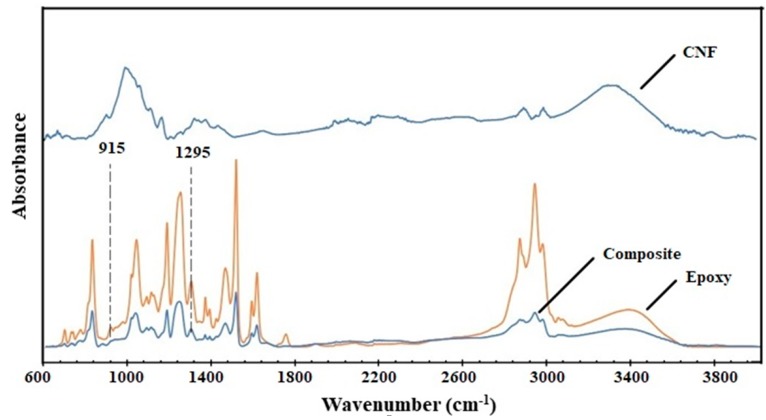
FTIR spectra representative of CNF, neat cured epoxy resin, and composites.

**Figure 6:**
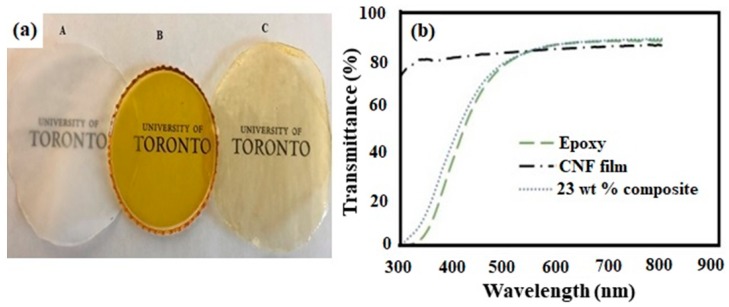
(**a**) Images of pure CNF (A), cured epoxy resin only (B), and the 23 wt % composite film (C). (**b**) Light transmittance measurements versus the wavelength for pure CNF, the cured epoxy resin, and the 23 wt % composite.

Figure 5 shows the infrared spectra of pure CNF film, cured neat epoxy, and composite with 23 wt % CNF. CNF film showed major absorption peaks at 3330 cm^−1^ (O-H stretching), 1161 cm^−1^ (C-O-C asymmetric stretching), 990 cm^−1^ and 1056 cm^−1^ (C-O stretching), 1369 cm^−1^ (symmetric C-H deformation), 1425 cm^−1^ (asymmetric C-H deformation) associated with various polysaccharides (cellulose and hemicellulose) [35,36]. The cured neat epoxy showed major peaks at 3370 cm^−1^ (O-H stretching), 1607 cm^−1^ (C=C stretching in phenyl groups), 1036 cm^−1^ (C-O symmetric stretching in ether groups), 1334 cm^−1^ (C-H deformation in epoxide groups), 1295 cm^−1^ (C-H vibration in epoxide groups), and 915 cm^−1^ (in-plane asymmetric deformation of epoxide ring) [35,37,38]. For composites, the infrared spectra were obtained on the polished surface. This was done mainly to remove the capping of epoxy layers on the surface (Figure 1c). In addition to hydroxyl peaks, there was significant weakening of peak intensities in the composite sample compared to epoxy, indicative of possible interaction between CNF and resin. The epoxide groups react with amines as well as the hydroxyl groups in CNF during curing [23,35]. Compared to cured epoxy, the composite samples showed a reduction in intensity or complete disappearance of absorption peaks at 1295 and 915 cm^−1^, related to the epoxide/oxirane rings in the resin. The CNF has high surface area with enormous amount of hydroxyl groups, which reacts with epoxides forming stable ether linkages [39]. In addition, several studies have highlighted the strong accelerating effect of celluloses or other hydroxyl groups on epoxy curing [37,40]. Figure 6 shows the images and the light transmittance measurements versus the wavelength for pure CNF, epoxy, and composite films. At 600–800 nm wavelength, the composite samples transmitted 86–88% of the light. The addition of uniform nano-sized cellulose fibrils in composites did not affect the light transmittance, when compared to neat epoxy films. The high light transmittance confirms the uniform dispersion of nanofibrils in the epoxy matrix [41].

**Figure 7:**
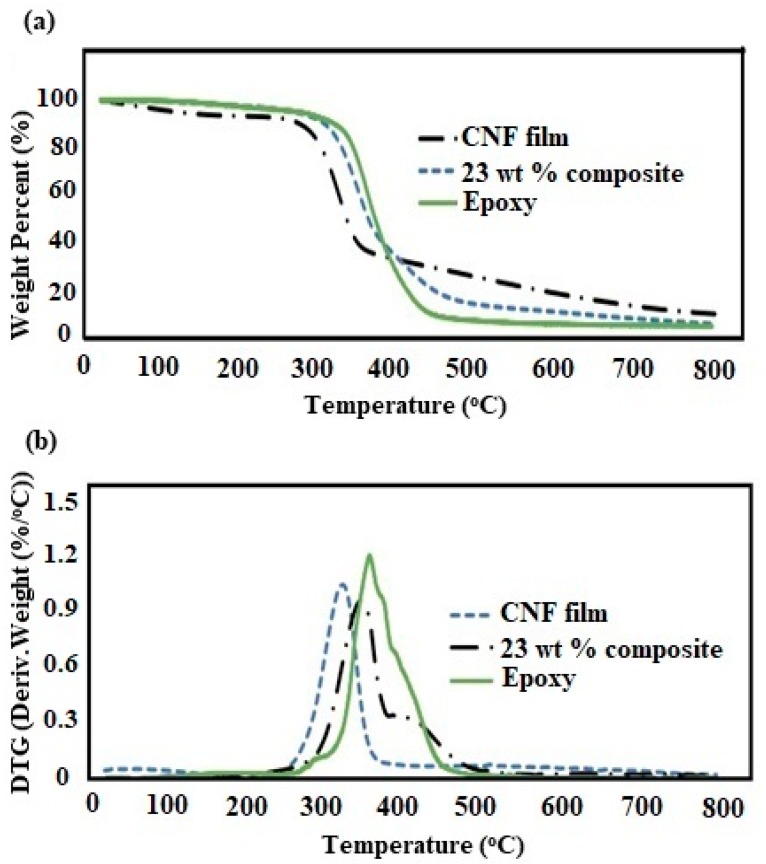
(**a**) TGA and (**b**) DTG curves for pure CNF, epoxy, and the 23 wt % composite.

**Figure 8:**
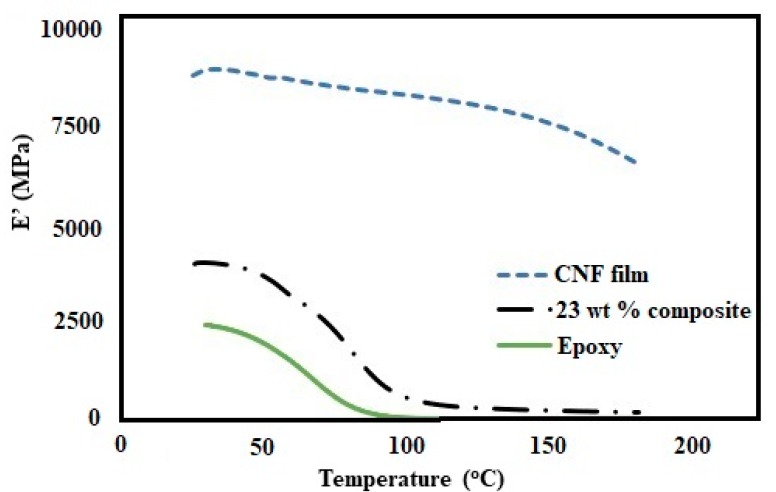
Temperature dependence of the storage modulus E′ for pure CNF, epoxy, and the 23 wt % composite.

**Figure 9:**
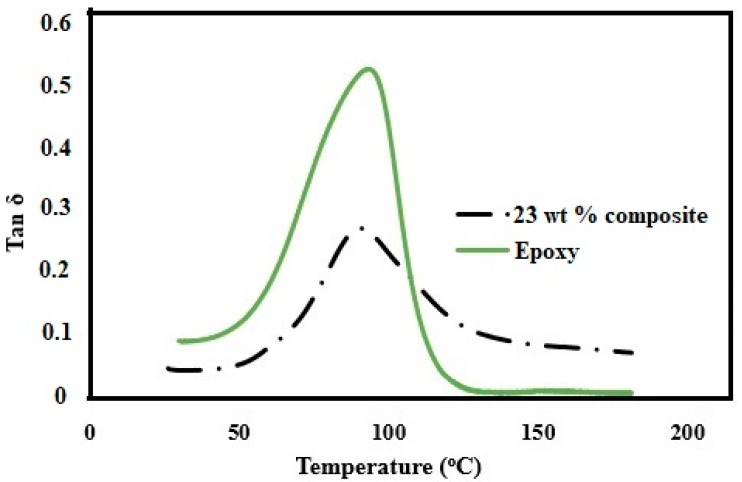
Temperature dependence of the tan δ for pure CNF, epoxy, and the 23 wt % composite.

Figure 7 shows the TGA and DTG curves for pure CNF, epoxy and 23 wt % composite. The pure CNF showed a T_onset_ and T_max_ of 286 and 321 °C, respectively. Despite being abundant and sustainable, the CNFs are still limited in composite industry mainly due to its low thermal stability [42]. However, epoxies are a class of thermosetting polymers with a high thermal stability. The epoxy resin used in our study showed a T_onset_ and T_max_ of 323 and 355 °C, respectively. The composites with 23 wt % CNF loading showed a T_onset_ and T_max_ of 308 and 345 °C, respectively. The decrease in T_onset_ and T_max_ was only 5% and 3%, respectively, in the composite compared to the pure epoxy. The results are impressive, given the fact that we have added high amount of cellulose nanofibrils in the composite. The high thermal stability for the composite can be attributed to effective interaction between fibrils and epoxy [23]. Figure 8 shows the storage modulus as a function of temperature for pure CNF, epoxy, and 23 wt % composite. The presence of CNF had a strong reinforcing effect in both glassy and glass transition region. The storage modulus significantly increased with the addition of 23 wt % CNFs. Figure 9 shows the tan δ as a function of temperature for epoxy and 23 wt % composite. The glass transition temperature (T_g_) at 91 °C did not show any significant difference between the epoxy and the composite. However, a significant decrease in intensity in tan δ peak for the epoxy matrix occurred with the addition of CNFs. The results imply that there was high interaction between fibrils and epoxy during the phase transition [25].

**Figure 10:**
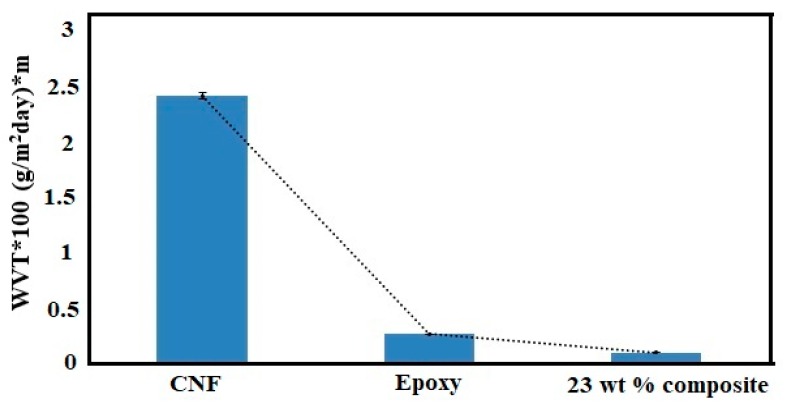
Water vapour transmission of the pure CNF, epoxy, and the 23 wt % composite.

Figure 10 shows the water vapour transmission of the neat epoxy, 23 wt % composite and the pure CNF film. The permeability of water vapour molecules was reduced by more than 60 % with the addition of CNFs to epoxy. The numerical values for WVT are given in Table S1. The water vapour permeability through polymer film depends on the dissolution of water molecules and its rate of diffusion in the film [27,43]. The diffusion of molecules in epoxy film takes place mainly through the interaction between the water molecules and epoxy matrix. Water molecules can hydrogen bond with either the hydroxyl groups or the amine groups in the resin [44]. The presence of hydroxyl groups in the cured epoxy was evident from the FTIR results. The water vapour barrier efficiency of pure CNF film is low mainly due to high hydrophilicity of cellulose molecules. However, the addition of highly crystalline, high aspect ratio CNFs can increase the tortuosity of permeating molecules within the epoxy matrix [27]. Different studies have showed that the water vapour efficiency of epoxy can be improved by using inorganic nanoparticles [45,46]. Water molecules can also diffuse through minute nano or micro voids within the film. Therefore, the high-water vapour barrier efficiency of composites also indicates the effective adhesion or interphase formation between the epoxy and CNFs. The WVT values we obtained for the composites can be compared to some of the films made from commercial petroleum-based polymers [27,47].

## 4. Conclusions

A strong reinforcement of CNFs on the epoxy polymer matrix was quite evident from different loading of CNFs (18–23 wt %). The average tensile modulus increased from 2.28 to 3.56 MPa, the tensile strength increased from 62.5 to 108.25 MPa, and the strain increased from 2.92% to 4.81%, which led to an overall increase in modulus of toughness by 184 times for the composites compared to the neat epoxy. The addition of fibrils also decreased the water vapor permeability of the neat epoxy, increasing the water vapour barrier efficiency of resulting composites by more than fifty percent. All these results indicate an effective interaction between the CNFs and epoxy used in this study. Also, the addition of substantial amount of cellulose fibrils (23 wt %) in the resulting composites did not affect the high thermal stability of the epoxy matrix. With improved mechanical properties, the commercial biobased epoxy resin reinforced with CNFs obtained in our study has great potential for various structural applications. The use of biobased epoxy and the addition of CNFs also highlight their environmental suitability. The low water vapour permeability obtained by the addition of CNFs in the epoxy are favorable attributes and can be extremely desirable for other applications such as coating or adhesives.

## Figures and Tables

**Table 1:** Mechanical properties of CNF film cured neat epoxy resin, and composites.

Sample	Tensile Strength (MPa)	Tensile Modulus (GPa)	Tensile Strain (%)	Strain Energy Density (MPa)
Pure CNF film	220 ± 21.7	7.7 ± 0.85	5.0 ± 0.47	7.53 ± 0.4
Epoxy	62.5 ± 5.86	2.28 ± 0.41	2.92 ± 0.33	0.98 ± 0.06
Composite (18–23 wt % CNF)	108.5 ± 8.43	3.56 ± 0.28	4.81 ± 1.04	2.79 ± 0.49

## Supplementary Materials

The following are available online at https://www.mdpi.com/2073-4360/11/4/612/s1, Table S1. Water vapour transmission of the epoxy, composite and the CNF film.
