# Supplementary material for: Highly Toughened and Transparent Biobased Epoxy Composites Reinforced with Cellulose Nanofibrils
Source: Polymers (Basel). 2019 Apr 3;11(4):612. doi: 10.3390/polym11040612 (PMC6523465; doi:10.3390/polym11040612)
Supplement: Supplementary file 1 [file polymers-11-00612-s001.pdf]

Supplementary information

Highly Toughened and Transparent Biobased Epoxy Composites Reinforced with Cellulose Nanofibrils

Sandeep S. Nair [1], Christopher Dartiailh [2], David B. Levin [2], Ning Yan [1,3] *

[1] Faculty of Forestry, University of Toronto, 33 Willcocks Street, Toronto, Ontario M5S 3B3, Canada;
[2] Department of Biosystems Engineering, University of Manitoba, E2-376 Engineering and Information Technology Complex (EITC), 75 Chancellors Circle, Winnipeg, MB R3T 5V6, Canada;
[3] Department of Chemical Engineering and Applied Chemistry, University of Toronto, 200 College Street, Toronto, Ontario M5S 3E5, Canada
* Correspondence: Tel.: +14169468070. Fax: +14169783834. E-mail: ning.yan@utoronto.ca.

Table S1. Water vapour transmission of the epoxy, composite and the CNF film.

Type of sample	WVT (g/m^2day)	Thickness (μm)	WVT *100 (Thickness normalized) (g/m^2day) *m
**CNF film**	536.73 ± 1.10	45 ± 0.66	2.42 ± 0.03
**Epoxy**	4.48 ± 0.41	600 ± 6.21	0.27 ± 0.00
**23 wt % composite**	2.99 ± 0.06	327 ± 2.11	0.10 ± 0.00
